# Long-term prognosis of Guillain-Barré syndrome not determined by treatment options?

**DOI:** 10.18632/oncotarget.20620

**Published:** 2017-09-01

**Authors:** Ying Wang, Wenjuan Lang, Yaqian Zhang, Xiaoyi Ma, Chunkui Zhou, Hong-Liang Zhang

**Affiliations:** ^1^ Department of Neurology, The First Hospital of Jilin University, Changchun, China; ^2^ Department of Neurology, The Affiliated Hospital to Changchun University of Chinese Medicine, Changchun, China; ^3^ Department of Life Sciences, The National Natural Science Foundation of China, Beijing, China

**Keywords:** Guillain-Barré syndrome, long-term prognosis, predictors, self-limitation, intravenous immunoglobulin

## Abstract

**Background:**

The long-term follow-up system for Guillain-Barré syndrome (GBS) is not well established worldwide. In our study, the preliminary data of the long-term prognosis of GBS are collected to explore the prognosis of GBS and the effect of intravenous immunoglobulin (IVIg) treatment.

**Methods:**

The follow-up data of 186 patients with GBS admitted from 2003 to 2013 were collected in 2015 *via* phone interview. The GBS disability scale score was ranked by clinician to evaluate the long-term prognosis. The clinical data during the acute phase were also collected.

**Results:**

The mortality rates were 2.15%, 5.45% and 7.89% at discharge, 2-5 years and 6-10 years after disease, respectively. The GBS disability scale score improved dramatically from discharge to 2-12 years after the acute phase. The self-limitation, the spontaneous recovery of disease, occurred both at acute phase and 2-5 years after discharge. Comparisons between IVIg-treated patients and GBS patients who only received supportive care revealed no significant difference of long-term prognosis.

**Conclusion:**

The long-term prognosis of GBS appears not to be influenced by treatment options. The long-term improvement of IVIg treated-patients might be due to the self-limitation of GBS per se instead of the IVIg treatment.

## INTRODUCTION

Although the short-term prognosis of Guillain-Barré syndrome (GBS) is well identified, the long-term follow-up system is not well established worldwide [1-4]. GBS patients are followed up within one year after discharge in most cases, and only a small amount of studies follow the patients more than one year [1-4]. For the short-term outcome of GBS, a one-year follow-up study based on 527 GBS patients demonstrated that the mortality rate within 12 months after onset was 3.9%, distributed to 20%, 13% and 67%, during the acute, plateau and recover phases respectively [1]. The prognostic factors for death were age, the severity of disease and the speed of progression [1]. For survivors, a rapid recovery was observed within the first year after the disease [2]. The proportion of GBS patients with complete recovery or minor limitations was 41% in the first month, 71% in the third, 86% in the sixth, and 92% in the twelfth [2]. A longer follow-up study revealed that the full functional recovery, minor deficits and aid-need occurred in 64%, 27% and 9% of all GBS patients within 3-5 years after the onset [3]. Reduced walking ability was present in 52% of 29 patients, 10 years after illness [4]. As for the predictors of the prognosis, the results of numerous studies are controversial. Except that one of them failed to find the predictors [3], most of the others identified distinct predictors of prognosis, including older age, severe disability at admission and nadir, cranial nerve involvement, ventilator dependence, absence of respiratory infections, autonomic dysfunction, neck flexor weakness and acute motor axonal neuropathy, and so forth [2-9]. Furthermore, a model of clinical prognostic scoring system was established to predict the prognosis of GBS [10, 11]. Age, antecedent diarrhea, GBS disability score 2 weeks after entry, and the Medical Research Council (MRC) sum score at hospital admission were considered as the main factors to assist the prediction of the outcome at 6 month [10, 11].

Supportive medical care and immunotherapy are two cornerstones of the management for GBS [12]. Intravenous immunoglobulins (IVIg) and plasma exchange are two most effective treatments while the use of steroids is not thought to be beneficial [12]. Six large comparative studies between IVIg and plasma exchange have been conducted on GBS patients, whereas plasma exchange and IVIg were found as equally effective [13, 14]. Of note are the flaws of the above-mentioned studies. Most of the follow-up studies last no more than one year after the disease. Moreover, comparative randomized controlled trials comparing IVIg and placebo/supportive care have not yet been performed [13], and some researchers have failed to include patients who exhibited a mild disease course in the trials [15].

Seemingly, a majority of patients with GBS experience a quick recovery within one year after the acute phase [2]. Thus, most of the follow-up studies are stopped at the first year after discharge, and the effect of IVIg is also explored based on the one-year follow-up data [13, 14]. However, residual deficits do exist among GBS patients [2]. Although most of the patients have a good outcome one year after discharge, not all of the patients recover completely within one year [2]. Herein, we explore the prognosis in a large cohort of GBS patients 2 to 12 years after the acute phase, and estimate the long-term effect of IVIg by comparing the long-term GBS disability scores in IVIg-treated patients *versus* those who had merely received supportive care.

## RESULTS

### Distinct mortality rates at different disease stages

**Figure 1:**
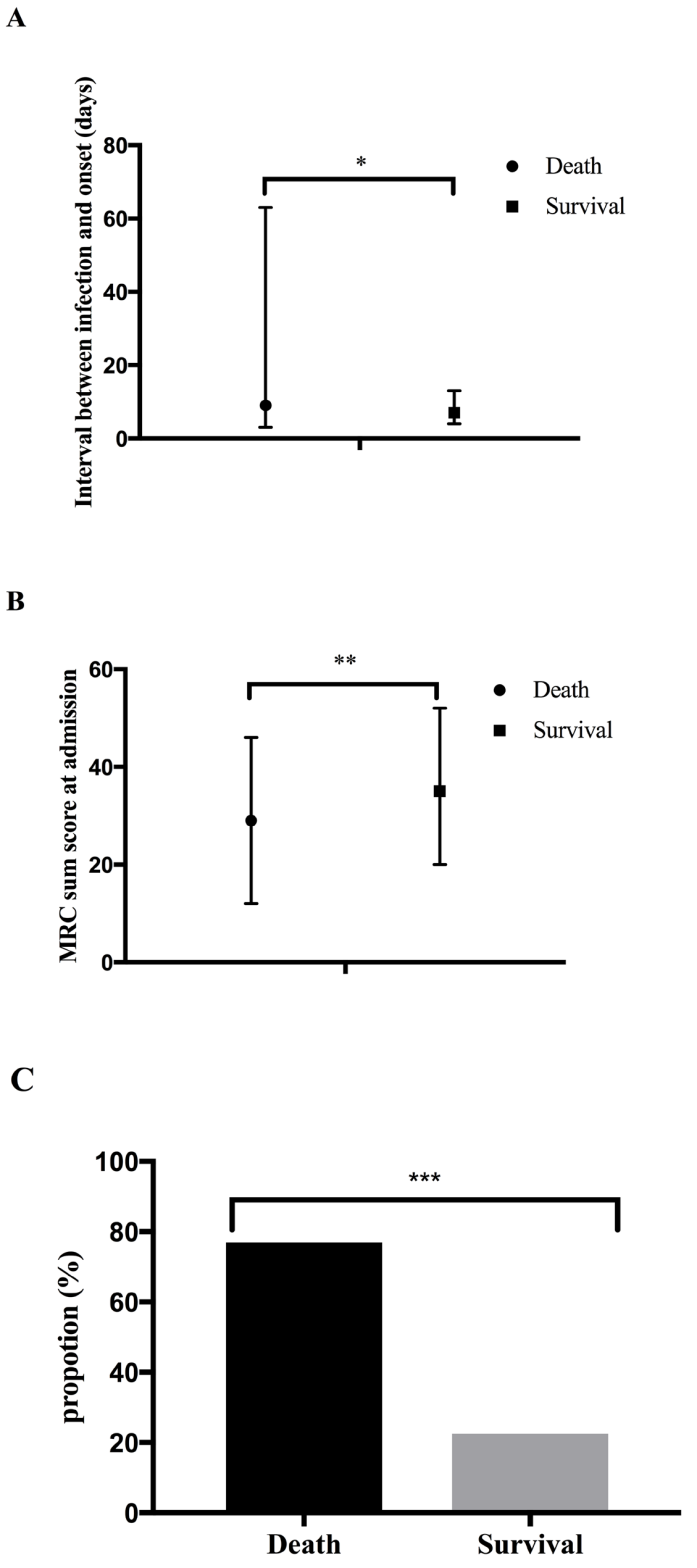
Predictors for death **A.** Predictors for death, including death occurring in the acute phase and death 2-12 years after discharge, were explored. The median of the interval between antecedent infection and disease onset was 9 days with IQR of 3-63.5 among death, while 7 days with IQR of 20.25-52.5, among survivals. **B.** The median of the MRC sum score at admission among death was significantly lower than among survivals (29 with IQR of 12-46.5 *vs* 35 with IQR of 20.25-52.5). **C.** About 76.92% of the death developed dyspnea during disease course, and its counterparts among survivals was 22.54%. *p < 0.05, **p < 0.01, *** *p* < 0.001.

Data of demographics, medical history, clinical manifestations, laboratory findings and treatment are presented in Table 1. One hundred and eighty-six enrolled patients were further divided into two groups. One-hundred and ten patients interviewed 2 to 5 years after the disease onset (admitted from 2010 to 2013) were designated into the 2-5 years group, and the other 76 patients interviewed 6 to 12 years after the onset (admitted from 2003 to 2009) fell into the 6-12 years group. The data were matched, and no significant difference of gender distrubution, age and disease severity between the two groups during hospitalization was noted (Table 2). During the acute phase, 2.15% of the admitted GBS patients died as a direct result of the disease. For death, cardiac arrest and respiratory failure were the common causes, which account for 50% and 50% of the causes respectively. The mortality rates were 5.45% and 7.89% in the 2-5 years group and the 6-12 years group respectively. A longer interval between antecedent infection and disease onset, lower MRC sum score at admission and dyspnea during the acute phase were identified as predictors of death (Figure 1A-1C).

**Table 1:** Description of enrolled GBS patients

Basic information	
Male/female ratio	108/78
Age (years) ^a^	40.5 (29.75-54.25)
Duration in hospital (days)^a^	14 (9-19)
Medical history	
Hypertension	17.20%
Diabetes	4.84%
Symptoms of antecedent infection	57.52%
Interval between infection and onset (days)^a^	5 (2-12)
Diarrhea	31.72%
Upper respiratory tract infection	29.03%
Clinical manifestations	
MRC sum score at admission ^a^	42 (35.5-52.5)
GBS disability scale score at admission ^a^	4 (2-4)
MRC sum score at nadir^a^	40 (30.75-48)
GBS disability scale score at nadir ^a^	4 (3-4)
Interval between onset and nadir (days) ^a^	6 (4-8)
MRC sum score at discharge ^a^	52 (44-60)
GBS disability scale score at discharge^a^	2 (1-4)
Decreased muscle tonus	26.88%
Cranial nerve involvement	45.16%
Hyporeflexia/areflexia	85.55%
Superficial sensation deficits	45.70%
Deep sensation deficits	5.91%
Autonomic deficits	57.53%
Dyspnea	26.34%
Ventilator dependence	15.05%
Complications during hospitalization	
Pulmonary infection	20.43%
Embolism	2.15%
Electrolyte disturbance	5.91%
Blood routine examination at admission	
White blood cell (*10^9^/L)^b^	9.14
Neutrophil (%)^b^	0.69
Lymphocyte (%)^b^	0.24
Lumbar puncture	
Protein concentration (g/L)^b^	0.64
White blood cell (10^6^/L)^a^	4 (2-7)
Albumin-cytologic dissociations	62.61%
IgG concentration (mg/L)^b^	100.33
Nerve conduction studies	
Demyelinating group	49.09%
Axonal group	30.90%
Overlap group	20.01%
Treatment	
IVIg	40.32%
Interval between onset and IVIg^a^	4 (3-7)
Glucocorticoids	13.44%
IVIg + Glucocorticoids	20.43%
Supportive care	25.81%

^a^Median (IQR)

^b^Average

**Table 2:** Comparison between patients out of touch and in touch

	In touch	Out of touch	*p* value
Gender (male/female)	120/85	252/158	0.484
Age(years) ^a^	40 (27.75-54)	37.5 (25-52.25)	0.080
The MRC sum score ^a^			
At admission ^a^	46 (36-54)	48 (36-56)	0.530
At nadir ^a^	42 (31.75-52)	46 (32-54)	0.305
At discharge ^a^	54 (43.75-58.5)	52 (42-60)	0.678

^a^Median (IQR)

### Gradual recovery 2-12 years after the acute phase

**Figure 2:**
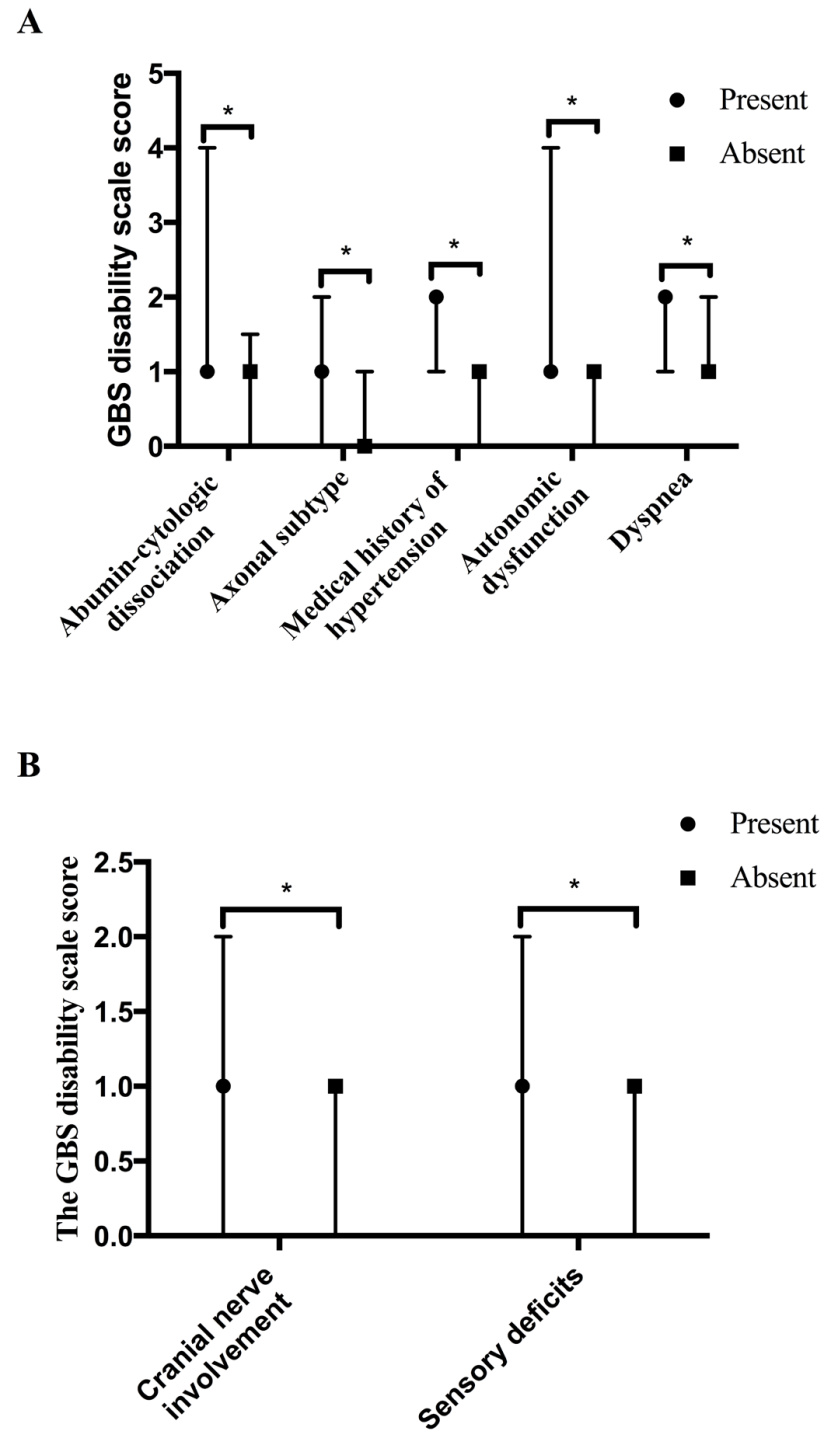
Predictors for 2-5/6-12-year outcome **A.** Predictors of the GBS disability scale score in 2-5 years group. GBS patients with appearance of abumin-cytologic dissociation (56.00%, 42/75), axonal subtype (53.12%, 17/32), medical history of hypertension (19.09%, 21/110), autonomic dysfunction (59.09%, 65/110) and dyspnea (28.18%, 31/110) had higher GBS disability scale scores than patients without these symptoms in the 2-5 years group (the averages of the GBS disability scale score: 1, 1, 2, 1 and 2, with IQR of 1-4, 0-2, 1-2, 1-4 and 1-2 *vs* 1, 0, 1, 1 and 1, with IQR of 0-1.5, 0-1, 0-1, 0-1 and 0-2). **B.** Predictors of the GBS disability scale score in the 6-12 years group. Cranial nerve involvement and sensory deficits were proved to predict the long-term prognosis 6-12 years after acute phase (average of GBS disability scale score, present: 1 and 1, with IQR of 0-2 and 0-2 *vs* absent: 1 and 1, with IQR of 0-1 and 0-1).

The medians of the GBS disability scale score at admission, nadir and discharge were showed in Table 1. The medians of the GBS disability scale score 2-5 years and 6-12 years after discharge were 1 and 0, with interquartile range (IQR) of 0-2 and 0-1, respectively. Although it was about 1 score lower in the 6-12 years group than in the 2-5 years group, no significant difference of the GBS disability scale score between two groups was observed (*p* = 0.222). The predictors for 2-5 years and 6-12 years prognosis were further analyzed. Appearance of albumino-cytologic dissociation, axonal subtype (including acute motor axonal neuropathy and acute motor sensory axonal neuropathy [12]), medical history of hypertension, autonomic dysfunction (defined by symptoms) and dyspnea correlated with a significant higher GBS disability scale score in the 2-5 years group, and were predictors for frustrated outcome 2-5 years after disease onset (Figure 2A). The GBS disability scale score in the 2-5 years group was correlated with the GBS disability scale score at discharge, the lymphocyte count in blood at admission, protein and IgG concentration in CSF as well as the MRC sum score at admission/nadir/discharge (*p* values: 0.002, 0.009, 0.007, 0.007, 0.006, 0.009 and 0.000; rs values: 0.304, -0.262, 0.309, 0.319, -0.262, -0.253 and -0.358). Cranial nerve involvement and sensory deficits found *via* physical examinations at admission predicted worse long-term prognosis 6-12 years after the acute phase (Figure 2B). The GBS disability scale score in the 6-12 years group was correlated with the interval between infection and onset and the GBS disability scale score at nadir and at discharge (*p* values: 0.025, 0.024 and 0.002; rs values: 0.347, 0.258 and 0.364).

### Self-limitation of the disease course of GBS

The self-limitation, the spontaneous recovery of disease course, of GBS was explored on patients who received supportive care only during hospitalization. Only supportive care was given if patients presented with a mild disease course or if they refused an immuno-modulatory therapy (unable to afford it in most circumstances). The medians of GBS disability scale score at admission/at nadir/at discharge/2-5 years/6-10 years, of patients who were given supportive care only, were 3, 3, 2, 0.5 and 0, with IQR of 2-4, 2-4, 1-4, 0-2 and 0-1, respectively. The data disclosed that the GBS disability scale score was grossly improved by 1 score from nadir to discharge, and another 1 score 2-5 years after discharge. The average improvement from nadir to discharge was 0.51 MRC sum score per day and 0.06 GBS disability scale score per day. As no significant difference of outcome between the 2-5 years group and the 6-12 years group among supportive care patients was observed (*p* = 0.222), the data of the two groups were combined to gain a larger sample volume in the subsequent analysis. The factors influencing the self-limitation process were studied. The self-limitation process was divided into short-term self-limitation (SS) and long-term self-limitation (LS) period, which were evaluated by SS score (the GBS disability scale score at nadir minus its counterpart at discharge) and LS score (the GBS disability scale score at discharge minus the one gained 2-12 years later). Patients who experienced antecedent diarrhea had a higher SS score (1, with IQR of 0-2 *vs* 0, with IQR of 0-0). Duration of hospitalization was positively correlated to the SS score (*p* = 0.001, *rs* = 0.259). Intervals from infection to onset and the MRC sum score at admission/nadir were negatively and positively correlated to the LS score (*p* values: 0.032, 0.001 and 0.000; rs values: -0.241, -0.318 and -0.320).

### No effect of IVIg on the long-term outcome of GBS

**Figure 3:**
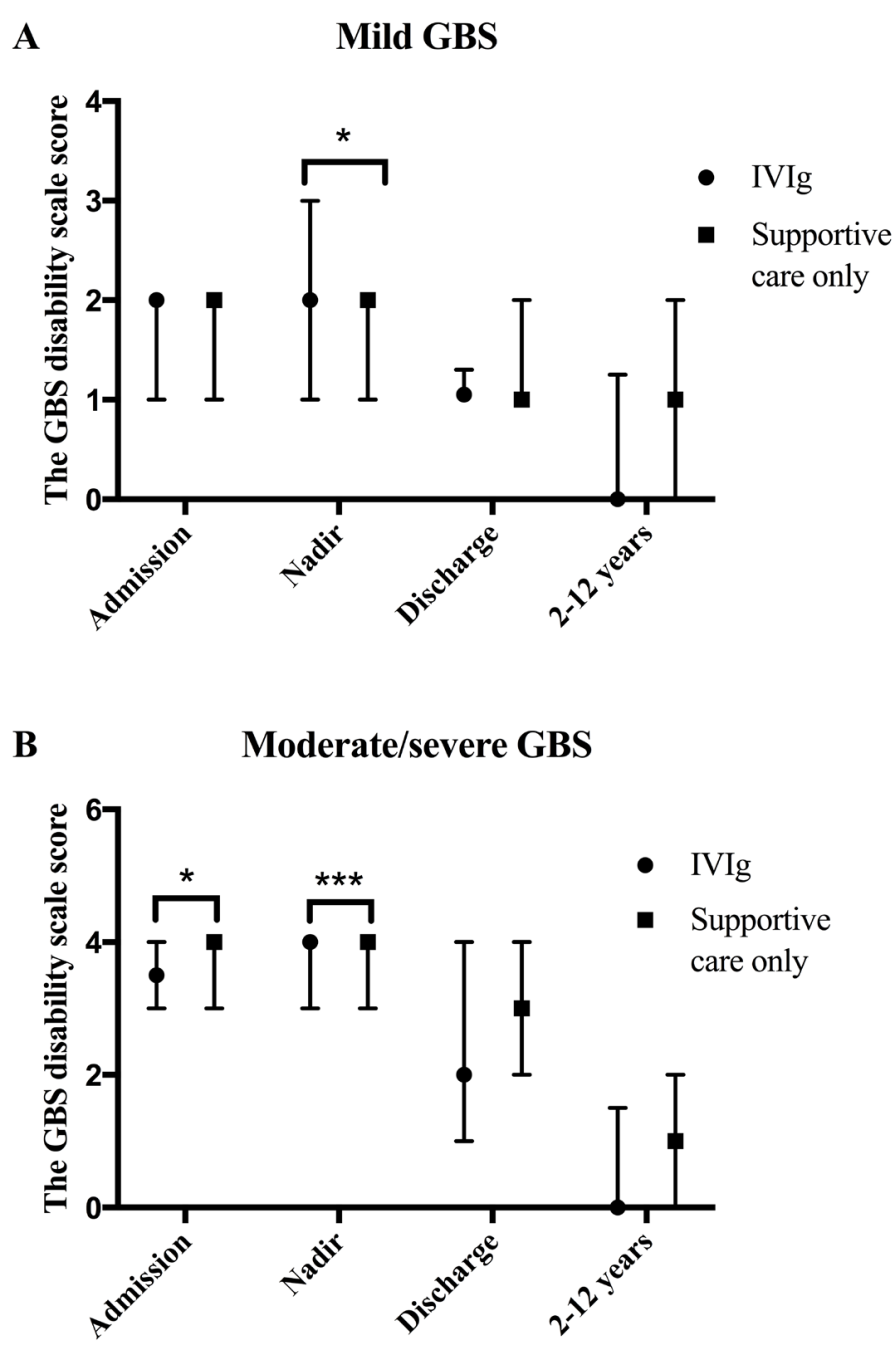
Effects of IVIg on GBS **A.** In the mild GBS group, the GBS disability scale scores of IVIg-treated patients and supportive care-received patients at admission, nadir, discharge and 2-12 years after the acute phase were 2, 2, 1, 0, with IQR of 1-2, 1-3, 1-1.25, 0-1.25 and 2, 2, 1, 1, with IQR of 1-2, 1-2, 1-2, 0-2, respectively. IVIg-treated patients had a significantly higher GBS disability scale score at nadir. **B.** In the moderate/severe GBS group, the GBS disability scale scores, of patients who were treated with IVIg, at admission, nadir, discharge and 2-12 years after the acute phase were 3.5, 4, 2, 0, with IQR of 3-4, 3-4, 1-4, 0-1.25, and its counterparts of GBS patients who were received supportive care only were 4, 4, 3, 1, with IQR of 3-4, 3-4, 2-4, 0-2, respectively. Patients in supportive care group had a significantly higher GBS disability scale score both at admission and at nadir.

Patients who received glucocorticoids were excluded. IVIg was the only immunotherapy for 213 out of all GBS patients during the acute phase, among whom 72 GBS patients who were able to be followed were divided into two groups to analyze the effect of IVIg (Table 3). Eighteen of them with GBS disability scale score less than 3 at nadir were considered as mild GBS, the other 54, with GBS disability scale score equal to or over 3, were regarded as moderate/severe GBS (Table 3). GBS patients who had supportive care only were also divided into two groups accordingly as negative controls (Table 3). No significant difference of the GBS disability scale score at 2-12 years was observed between the supportive care group (mild GBS) and the IVIg group (mild GBS) as well as the supportive care group (moderate/severe GBS) and the IVIg group (moderate/severe GBS) (Figure 3A and 3B). No evidence implied that the IVIg treatment could significantly improve the long-term prognosis.

**Table 3:** Description of GBS patients with mild and moderate/severe disease courses

Groups	Mlid GBS		Moderate/severe GBS	
Management	IVIg	Supportive care	IVIg	Supportive care
Admission, nadir and discharge (number, male/female)	54, 37/17	40, 23/17	159, 87/72	78, 44/34
2-12 years (number, male/female)	18, 11/7	17, 12/5	54, 26/28	27, 18/9

## DISCUSSION

The prognosis of GBS is favorable, and most of the follow-up studies have identified the outcome of GBS patients one year after the acute phase [1, 2]. However, many of the victims are left with residual disability [1-4], and the long-term follow-up system is not well established worldwide. We have explored the long-term prognosis of GBS and the influence of IVIg treatment. We found that the mortality rates of GBS differed in distinct disease stages. The recovery phase of GBS was as long as two to five years after discharge. Self-limitation of the disease course occurred from the acute phase to 2-5 years after discharge. IVIg treatment did not contribute to a favorable long-term prognosis, and the long-term recovery may be due to the self-limitation of the disease course.

Limited studies on long-term prognosis of GBS are available. Forsberg et al identified reduced walking ability and facial paralysis as residual symptoms of GBS patients 10 years after disease onset [4]. However, only 29 subjects were enrolled in their study [4]. Bersano et al followed 70 GBS patients 3-5 years after disease onset *via* phone interviews, and found that 36% of the patients had residual symptoms [3]. When compared to their research, our study had a larger sample volume and longer follow-up period. The reported mortality rate of GBS after discharge varied among studies [1, 5, 16]. According to our results, the mortality rate of GBS was increased from discharge to 6-12 years. This finding is in line with a previous study demonstrating that the majority of the death occurred in the recovery phase [1]. Self-limitation of the disease course has long been indicated in GBS [12, 17, 18]. Mice with experimental autoimmune neuritis, a widely used animal model of GBS, started to develop paralysis approximately 7 days post immunization with P0 peptide 180-199, reached nadir 28 days post immunization, and subsquently entered a spontaneous recovery period [17]. Typically, a monophasic disease course occurred in GBS patients, including the progressive stage, plateau and the recovery phase [18]. However, this retrospective study was conducted on GBS patients who received immunomodulatory therapy, which could influence the process of self-limitation. In our study, the self-limitation of GBS was further confirmed in the supportive care group. Patients with GBS recovered spontaneously from nadir to discharge and from discharge to the forthcoming 2-5 years. Associations between the prognosis and the treatment modalities of GBS have been studied; however, several flaws are notable. Few comparative studies on treatment effect were performed between the IVIg group and the placebo/supportive care group [13]. Investigations have been focused on effect of IVIg on moderate/severe GBS patients who are unable to walk 10m unaided [12, 19], but mild GBS patients with the GBS disability scale score less than three are ignored to some extent. Moreover, studies on the long-term impact of IVIg on GBS patients are rarely conducted. We subsequently explored the effect of IVIg among both mild and severe/moderate GBS patients in comparison with matched supportive care group. Surprisingly, both of the long-term outcomes were not different from the IVIg group and the supportive care group. The potential explanations are herein discussed. Firstly, in the history of studying immunomodulatory therapy for GBS, plasma exchange is the first milestone. The first randomized trial of plasma exchange *versus* supportive care in 1984 yielded a negative result [20]. However, some similar studies conducted subsequently obtained positive results [13, 21]. When IVIg therapy emerged, most of the investigators studied the effect of IVIg *via* leading comparative researches between IVIg and plasma exchange on severe GBS patients [13]. Most studies showed no significant difference of the effect between IVIg and plasma exchange. Thus, IVIg is thought to be effective. Specifically, the influence of the self-limitation of the disease course is not taken into consideration. Secondly, the outcome of GBS may be largely decided by the ability of repairing rather than the severity of injury. IVIg is reported to play a role of attenuating immune response by inhibiting Fc-mediated activation of immune cell, attack to peripheral nervous system mediated by antibodies as well as complements [12]. It means that IVIg does not contribute to the repairing process. Actually, the mechanisms of the long-term repairing process of GBS remain unknown.

Our study has limitations. Firstly, the main data were acquired *via* phone interviews, and only the GBS disability scale was used to evaluate the long-term prognosis. As mentioned above, the long-term follow-up system is not well established worldwide. It usually will take around 10 years and great efforts to carry out a well-designed study to explore the long-term prognosis of GBS. In addition, many of the patients may drop out from long-term follow-up studies, because most of them may have a good outcome. Although evaluating the long-term prognosis *via* acquiring the GBS disability scale score by phone interview was not perfect, the data were still powerful. The GBS disability scale score, which is based on the ability of patients to run and to walk [22], is a widely used scale to evaluate the disease severity during the acute phase. Even *via* phone interview, it was not hard for patients to answer the questions exactly, such as “Are you able to run currently?”, “How long could you walk without assistance?”, etc. The data are what is available currently, and our preliminary data have filled in the blank of the long-term prognosis of GBS and long-term effect of IVIg on GBS patients to some extent. Secondly, 410 patients were out of touch due to the change of phone number or address, and the results may be influenced. However, we have showed that there was no significant difference in disease severity during the acute phase between the patients who were able to be followed up, and patients who were out of touch. Thirdly, a few patients with acute-onset chronic inflammatory demyelinating polyradiculoneuropathy might have been enrolled due to the inadequate follow-up data [23]. Additionally, it is hard to discriminate if the death of the patients is related to GBS 2-12 years after discharge, thus the data of long-term mortality rate may be biased. Finally, the self-limitation of the disease course was only proved on the patients with a relatively mild disease course.

In summary, we have conducted a study on the long-term effect of IVIg treatment on both mild and moderate/severe GBS patients, and find that the outcome seems not determined by treatment options. The long-term improvement on IVIg-treated patients may be due to the self-limitation of the illness instead of the IVIg treatment. For further investigations, a long-term follow-up system is warranted for GBS patients. The long-term prognosis of GBS and the long-term effect of IVIg are needed to be analyzed according to different subtypes.

## MATERIALS AND METHODS

This study was approved by the Ethics Committee of the First Hospital of Jilin University. Though written informed consent was not obtained, patient information was de-identified.

### Study subjects

**Figure 4:**
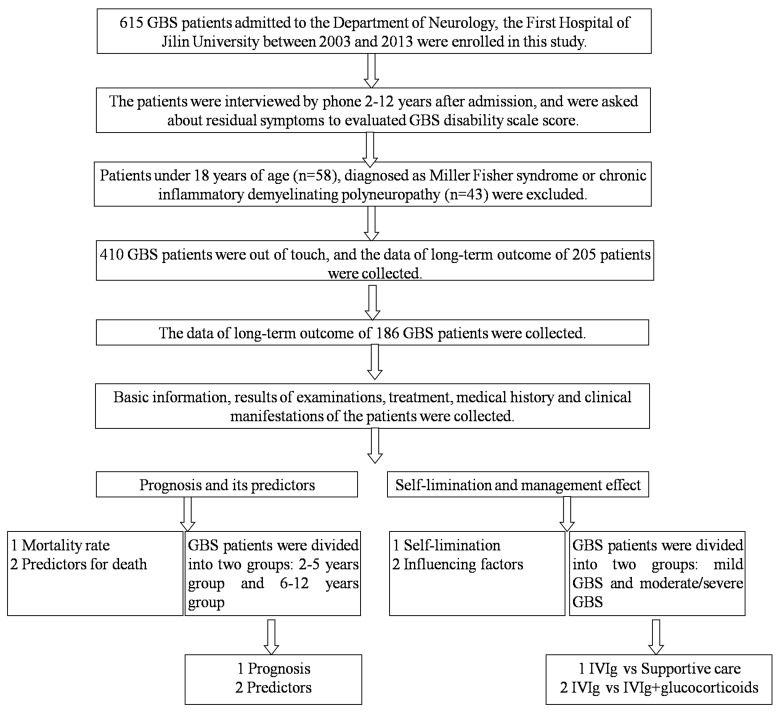
Flow chart of subject enrollment This study was based on a database comprising 615 consecutive GBS patients. Patients under 18 years of age, diagnosed as Miller Fisher syndrome or chronic inflammatory demyelinating polyneuropathy, and who could not be reached were excluded. 186 GBS patients were finally included for data analysis.

A cohort of 615 consecutive GBS patients fulfilling the standard diagnostic criteria [24], admitted from 2003 to 2013, were enrolled in this study. The patients were interviewed by telephone in 2015 (2 to 12 years after the initial admission), and were asked about residual symptoms. As the clinical features of patients less than 18 years of age were different from the adult patients, 58 children were excluded from our study [25]. Thirty-four patients, diagnosed with Miller Fisher syndrome or chronic inflammatory demyelinating polyradiculoneuropathy, were also ruled out. In the past decades, urbanization has greatly changed the life style of Chinese. For example, mobile phones have largely replaced home phones while a real-name system has not been established; people changed their mobile phone numbers easily. Meanwhile, the use of post codes is drastically declining. A total of 410 patients were out of touch most probably due to their change of mobile phone numbers. The data of long-term prognosis, demographics, medical history, clinical manifestations, laboratory findings and treatment from 186 followed patients were collected and analyzed (Figure 4). No significant differences of demographics, the MRC sum score at admission, at nadir and at discharge, were observed between the patients out of touch and patients who were able to be followed (Table 3).

### Evaluation of disease severity and functional impairments

The long-term prognosis of GBS patients was measured by the GBS disability scale, which was defined as follows: 0: healthy state; 1: minor symptoms and capable of running; 2: able to walk 5 meters or more without assistance but unable to run; 3: able to walk 5 meters across an open space with help; 4: bedridden or chair-bound; 5: requiring assisted ventilation for at least part of the day; 6: dead [22]. Besides, the severity of patients at admission, nadir and discharge was also described by the MRC sum score that could express the weakness of six bilateral muscles in arms and legs, ranging from 0 (tetraplegic) to 60 (normal strength) [26]. The lowest MRC sum score during the disease course was identified as the nadir of GBS [22, 26].

### Statistical analysis

Statistical analysis was performed by SPSS version 18.0 software (SPSS, IBM, West Grove, PA, USA). Differences in proportion, normal continuous variable and qualitative variable were tested by Chi-square or Fisher exact tests, student-t test and Mann-Whitney U test, respectively. Kruskal-Wallis test was used to compare values among groups followed by Mann-Whitney U test to compare values between groups. Correlations were tested by the Pearson rank correlation coefficient (r) or the Spearman rank correlation coefficient (rs). A two-side *p* value < 0.05 was considered as statistically significant for all statistical tests.

## Abbreviations

GBS: Guillain-Barré syndrome
IQR: interquartile range
IVIg: intravenous immunoglobulin
LS: long-term self-limitation
MRC: Medical Research Council
SS: short-term self-limitation
